# SARS-CoV-2 detection in primary thyroid sarcoma: coincidence or interaction?

**DOI:** 10.1007/s40618-021-01722-1

**Published:** 2022-01-05

**Authors:** M. L. Tanda, S. Ippolito, D. Gallo, A. Baj, F. Novazzi, A. Genoni, M. Annoni, N. Mancini, N. Clementi, G. Finzi, E. Piantanida, P. Premoli, A. Lai, D. Dalla Gasperina, F. Maggi, S. Uccella

**Affiliations:** 1 Department of Medicine and Surgery, University of Insubria, Varese, Italy; 2 Endocrine Unit, Department of Medicine and Surgery, University of Insubria, ASST Dei Sette Laghi, Viale Borri, 57, 21100 Varese, Italy; 3 Laboratory of Microbiology, ASST Dei Sette Laghi, Varese, Italy; 4 Endocrine Metabolic Surgery, ASST Dei SetteLaghi, Varese, Italy; 5 Laboratory of Microbiology and Virology, University Vita-Salute San Raffaele, Milan, Italy; 6 Pathology Unit, ASST Dei Sette Laghi, Varese, Italy

**Keywords:** SARS-CoV-2, Sarcoma, Thyroid, MDM2, Subacute thyroiditis

## Abstract

**Introduction:**

Thyroid dysfunctions associated with SARS-CoV-2 are emerging in scientific literature. During the second COVID-19 epidemic spread, we evaluated a patient with the suspect of subacute thyroiditis.

**Methods and results:**

Specimen from fine-needle aspiration of a hypoechoic undefined area was analyzed for cytology and for SARS-CoV-2 detection. SARS-CoV-2 was retrieved by real-time polymerase chain reaction on the cytologic sample, which was then cultured on Vero E6 cells and demonstrated to be cytopathic. Whole-genome sequence was deposited. Histological exam diagnosed a rare case of primary thyroid sarcoma with diffuse and strong expression of mouse double minute 2 homolog (MDM2) oncoprotein. Ultrastructural examination confirmed, in several neoplastic cells, the presence of viral particles in cytoplasmic vacuoles.

**Conclusions:**

In our hypothesis, SARS-CoV-2 and sarcoma coexistence could represent a synergistic interplay, ultimately favoring both viral persistence and tumor proliferation: the overexpression of MDM2 in tumor cells might have generated a favorable immunological niche for SARS-CoV-2 localization and, in turn, SARS-CoV-2 could have favored tumor growth by inducing MDM2-mediated p53 downregulation. Functional studies are needed to confirm this suggestive pathway.

We report here the first description of intrathyroidal localization of SARS-CoV-2 in a rare case of primary thyroid sarcoma, a histotype accounting for less than 1.5% of thyroid cancers [1]. The detected virus underwent molecular typing and culture, where it demonstrated cytopathogenicity.

Several thyroid diseases have been recently associated with SARS-CoV-2, including subacute thyroiditis [2], Graves’ disease and Hashimoto’s thyroiditis, hypothyroidism, as well as nonthyroidal illness syndrome [3]. Virus is thought to enter thyroid cells through SARS-CoV-2 receptor angiotensin-converting enzyme 2 (ACE2) and co-receptor Transmembrane Serine Protease 2 (TMPRSS2), both highly represented in organs of the endocrine system, such as the reproductive system, pancreas, and thyroid; also in addition, mRNA expression has been demonstrated in thyroid follicular cells [4]. Recently, SARS-CoV-2 RNA has been isolated from autoptic thyroid tissues from patients who died of COVID-19 [5]; interestingly, this localized infection is associated with an intense activation of the ﻿innate immune response, due to upregulation of ﻿ type I and type II interferon signaling [6], which could explain some of the clinical manifestations of thyroid dysfunctions secondary to SARS-CoV-2 infection.

As part of ongoing studies of SARS-CoV-2 detection in extra-pulmonary sites, cytologic samples of thyroid tissues were evaluated in our Center through Real Time-Polymerase Chain Reaction (rtPCR).

A 56-year-old male outpatient was admitted to our center in January 2021, during the second COVID-19 epidemic spread in Northern Italy, for a sudden onset of pain and compressive symptoms in the anterior neck suggestive for subacute thyroiditis or neoplastic mass. Neck ultrasound revealed a single 35 × 50 mm hypoechoic nodular area of the left thyroid lobe showing central vascularization in the context of an otherwise normal gland. Thyroid function tests proved a euthyroid state and negative antibodies. Fine needle aspiration (FNA) was performed, and cytomorphologic features of the nodule were conclusive for undifferentiated thyroid carcinoma. SARS-CoV-2 RNA was identified on FNA sample by rt-PCR (m2000 Abbott) and then used to successfully infect Vero E6 cells. Extracted RNA was sequenced, genomic reconstruction was performed and deposited as hCoV-19/Italy/LOM-UnINSU2/2021 (virus isolation, sequencing methods, and variant characteristics are detailed below). The pre-operative nasopharyngeal swab and blood sample tested for SARS-CoV-2 RNA and IgG antibodies (LIAISON® SARS-CoV-2 S1-S2, DiaSorin, Saluggia, Italy) were both negative. The patient had no symptoms consistent with acute SARS-CoV-2 infection, did not report previous coronavirus disease (COVID-19), and had not yet received the COVID-19 vaccine.

Total thyroidectomy was not feasible due to a baseline reduced signal at the intraoperative nerve monitoring, thus, a left thyroid lobe resection was performed. The gross evaluation identified a bulky white and lardaceous tumor, involving the whole left lobe, infiltrating anteriorly the cervical muscles and posteriorly the lower larynx and trachea, with full entrapment of the laryngeal nerve, not dissectible from the tumor; no evidence of lymph nodes involvement was found. Staging F-fluorodeoxyglucose positron emission tomography/computed tomography (FDG-PET/CT) did not identify other suspect sites for tumor localization.

Histological examination (Fig. 1A–B) of the lesion revealed a highly cellular proliferation (mitotic index 20 × 10 high power fields at × 400 magnification) of markedly atypical spindle, oval and anaplastic cells, arranged in irregular fascicles. At immunohistochemistry, neoplastic cells were negative for all epithelial markers, as well as for thyroid-specific markers. Neoplastic cells intensively expressed desmin (Fig. 1C) and CD34 (Fig. 1D) and showed partial nuclear stain for myogenin (Fig. 1E) and myogenic determination factor 1 (MYOD1) (Fig. 1F). S100 protein was only faintly expressed in a small number of cells, but murine double minute 2 (MDM2) was diffusely and strongly expressed (Fig. 1G), as also confirmed by the amplification of its locus shown by fluorescent in situ hybridization (not shown). All endothelial markers, except for CD34 were negative. Electron microscopy analysis revealed poorly differentiated mesenchymal cells with cytoplasmatic Weibel-Palade bodies (Fig. 1I). Based on of these findings, a diagnosis of high-grade sarcoma with partial differentiation to rhabdomyosarcomatous, angiosarcomatous and liposarcomatous features was made. Noteworthy, the ultrastructural examination showed, in several neoplastic cells, the presence of viral particles in cytoplasmic vacuoles (Fig. 1L).

**Fig. 1:**
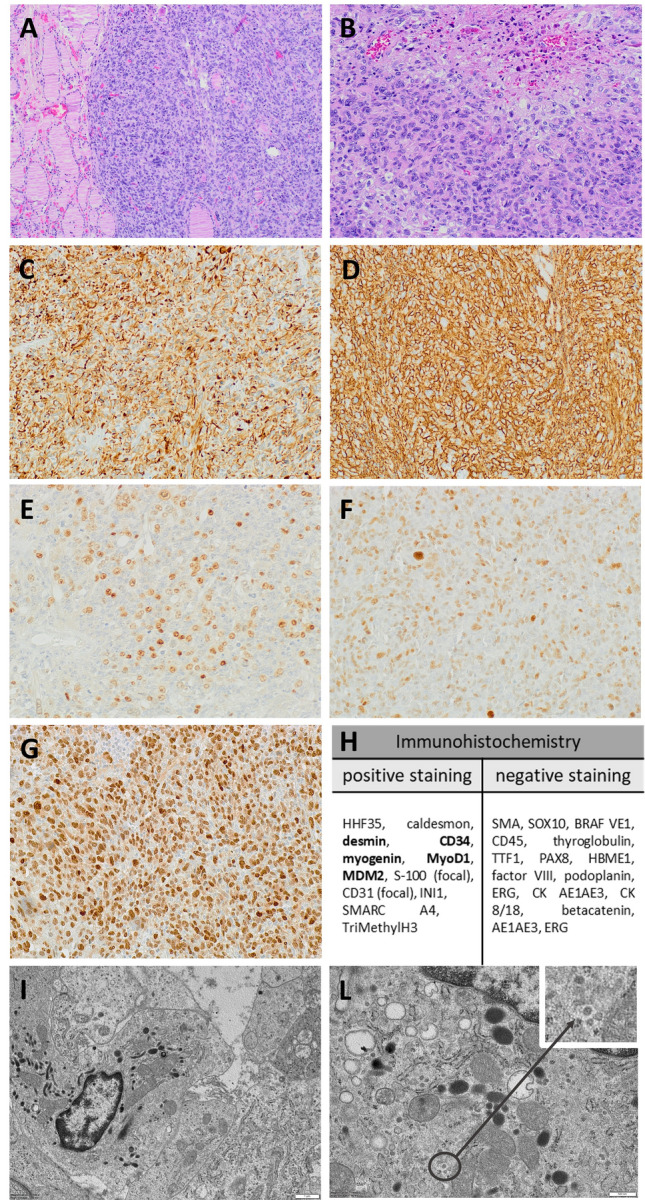
Histopathological and immunohistochemical features of the thyroid sarcoma. **A** Hematoxylin /Eosin (**H**&**E**): A hypercellular proliferation of atypical cells arranged in poorly defined fascicles effaces the normal thyroid parenchyma. **B** H&E: At higher magnification, spindle, oval and anaplastic cells with evident nuclear atypia are observed. A focus of tumor necrosis is present (top right). Immunohistochemical stains shows diffuse immunoreactivity for **C** desmin, **D** CD34, **E** myogenin, **F** MYO D1, and **G** MDM2. **I** Electron microscopy highlights Weibel-Palade bodies in neoplastic cells, proving an endothelial differentiation. **L** Viral particles included in intracytoplasmic vacuoles of neoplastic cells

Although we have soundly demonstrated SARS-CoV-2 localization in patient’s sarcomatous cells, we cannot rule out that this finding is incidental and has no clinical consequences. In our hypothesis, however, SARS-CoV-2 and sarcoma coexistence might represent a synergistic interplay, ultimately favoring both viral persistence and tumor proliferation. Sarcoma cancer cells displayed intense MDM2 immunostaining confirmed by FISH analysis, which showed MDM2 gene amplification. MDM2 oncoprotein is the principal negative regulator of p53. Besides its oncosuppressor activity, p53 is a pleiotropic molecule also related to antiviral-innate-immune responses, by inducing apoptosis of infected cells and mediating interferon production/signaling [7]. Thus, MDM2 and p53 pathways have been proposed as actors in COVID-19 development and cytokines storm, a hypothesis enforced by the proposal that SARS-CoV-2 papain-like-proteases (PLP) could be able to act as an MDM2 stabilizer, as proved for SARS-CoV-1 PLP [8], which has 86% amino-acid homology with SARS-COV-2 PLP [9]. The pathogenic hypothesis we hereby propose to explain this peculiar finding is presented in Fig. 2: the overexpression of MDM2 in tumor cells might have generated a favorable environment for SARS-CoV-2 infection which, in turn, might have caused MDM2 stabilization and p53 degradation, favoring tumor growth. To support this potential pathway, small molecules antagonizing MDM2, mining its interaction with p53, have been proposed both as a therapeutic agent for sarcomas as well as, more recently, to treat COVID-19 patients [10]. This peculiar niche, favoring virus infection and survival, may explain why nasopharyngeal swabs and subsequent serology were negative.

**Fig. 2:**
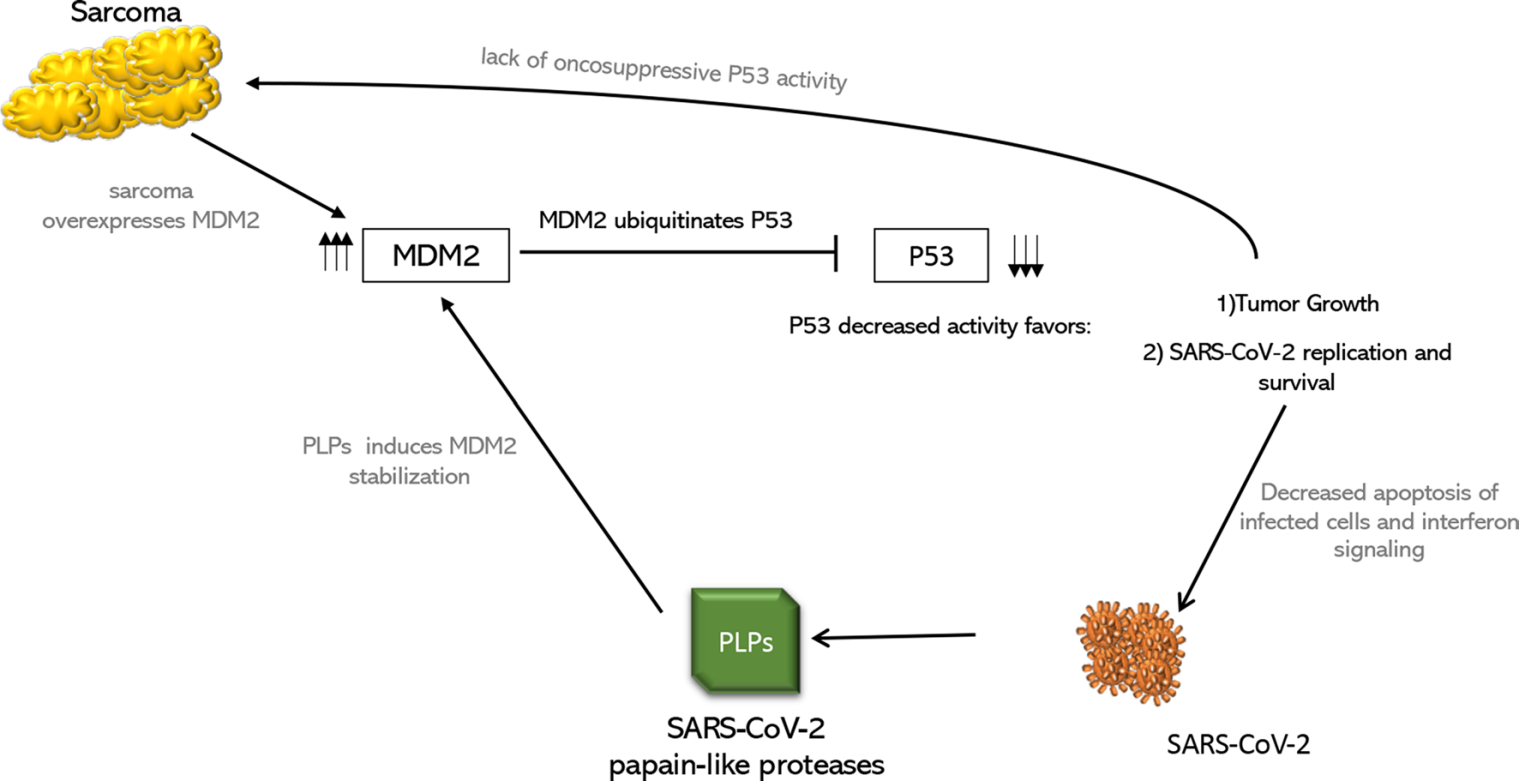
Proposed pathogenic pathway of the interplay between SARS-CoV-2 and sarcoma. Sarcoma and SARS-CoV-2 polylipoproteins (PLPs) concur to the overexpression of MDM2, which ubiquitinates p53. P53 downregulation, in turn, favors cancer proliferation and SARS-CoV-2 infection and survival

In an alternative or complementary scenario, SARS-CoV-2 infection might have primarily induced tumorigenesis due to (i) SARS-CoV-2 indirect oncogenic effect, via unproper stimulation of pro-inflammatory immune response and intracellular signaling, ultimately favoring oxidative stress and tumor growth; (ii) SARS-CoV-2 interaction with DNA of infected cells via the LINE-1 retrotransposition machinery [11]. Relatedly, polyomavirus simian virus 40 (SV40) has been retrieved at high prevalence in thyroid specimens of both differentiated and anaplastic thyroid cancer [12, 13]; SV40 proposed oncogenic mechanism involves a direct cooperation with host genome, activating oncogenes and growth factors expression. We overall find this hypothesis less plausible because (i) oncogenetic viruses are usually capable to integrate into the host DNA, which has been hypothesized for SV40 but not demonstrated for SARS-CoV-2 [14]; (ii) there is no current evidence of a massive increase in cancer diagnosis since the pandemic onset; (iii) viral sequence found in our patient seems attributable to a relatively recent variant, thus it is less likely that viral infection primarily triggered oncogenesis.

In conclusion, we have reported the first case of SARS-CoV-2 presence in a thyroid sarcoma arisen in a seronegative patient, who did not display any manifestation of the disease. To the best of our knowledge, this is the first case reporting SARS-CoV-2 presence in neoplastic cells. This novel finding sheds a light on the interplay between thyroid and SARS-CoV-2 and reinforces the hypothesis of a viral localization in thyroid tissue, which could explain the previous reported thyroid dysfunctions. Further research is needed to improve our understanding of active SARS-CoV-2 pathogenetic impact on thyroid gland and its possible interaction with cancerous cells.

## Virus isolation and sequencing

Vero E6 (Vero C1008, clone E6—CRL‐1586; ATCC) cells were cultured in Dulbecco's modified Eagle's medium (DMEM) supplemented with non‐essential amino acids (NEAA), penicillin/streptomycin (P/S), HEPES buffer, and 10% (v/v) fetal bovine serum (FBS). An aliquot (0.8 mL) of wash testing from FNA was mixed 1:1 with DMEM without FBS and supplemented with P/S and Amphotericin B. The mixture was added to 80% confluent Vero E6 cells monolayer seeded into a 25 cm^2^ tissue culture flask. After one hour adsorption at 37 °C, 3 mL of DMEM supplemented with 2% FBS and Amphotericin B were added. One day-post-infection (dpi), the monolayer was washed in PBS, and 4 mL of DMEM supplemented with 2% FBS and Amphotericin B were added. The cytopathic effect was monitored in inverted phase-contrast microscopy (Olympus CKX41) and the supernatant collected at monolayer complete disruption (3 dpi). The sample was heat-inactivated at 56 °C for 30 min, and the viral genome was extracted using QIAamp Viral RNA Mini Kit following the manufacturers' instructions (Qiagen). Extracted RNA was processed with the CleanPlex® SARS-CoV-2 Panel (Paragon Genomics) and sequenced with MiSeq Reagent Kit v2 (300-cycles) (Illumina, San Diego, CA, USA) on the MiSeq platform. Genomic reconstruction was performed using the SOPHiA DDM™ platform (SoPHiA Genetics).

Whole-genome sequence was deposited as hCoV-19/Italy/LOM-UnINSU2/2021; GISAID code: ID EPI_ISL_2007588. Type: betacoronavirus Clade GV Pango Lineage B.1.177 (lineages version 2021–06-05). AA Substitutions: Spike A222V, Spike D614G, Spike V308L, N A220V, NSP2 R380C, NSP3 S1437F, NSP12 P323L, NSP15 Q19R Variant.

This is a previously described SARS-CoV-2 uncommon variant, apparently devoid of peculiar characteristics or distinctive clinical relevance.
